# Medical service utilization and out-of-pocket spending among near-poor National Health Insurance members in South Korea

**DOI:** 10.1186/s12913-021-06881-8

**Published:** 2021-08-28

**Authors:** Sooyeol Park

**Affiliations:** 1 Division of Health Care Management and Policy, Department of Public Health Sciences, Graduate School of Public Health, Seoul National University, Seoul, 08826 Republic of Korea; 2 Department of Health Policy and Management, Johns Hopkins Bloomberg School of Public Health, 615 N. Wolfe Street, Baltimore, MD 21218 USA

**Keywords:** Medical utilization, Out-of-pocket spending, Catastrophic health expenditure, Poverty, Relative poverty

## Abstract

**Background:**

The public health care system in South Korea is a two-tiered system. The lowest-income population is covered by the Medical Aid program, and the remaining population is covered by the National Health Insurance. The near poor, a relatively low-income population which is excluded from South Korea’s Medical Aid program due to exceeding the income threshold, experiences insufficient use of medical services and incurs high out-of-pocket expenses due to a lack of coverage under the country’s National Health Insurance (NHI) program. This study aims to examine medical utilization, out-of-pocket spending, and the occurrence of catastrophic health expenditures among the near-poor group compared to both Medical Aid beneficiaries and other (higher income) NHI members.

**Methods:**

A cross-sectional study was conducted drawing upon a nationally representative dataset derived from the 2018 Korea Welfare Panel Study. The study classified people into three groups: Medical Aid beneficiaries; the near-poor population below 50 % of the median income threshold but still not qualifying for Medical Aid and thus enrolled in NHI; and NHI members above the threshold of 50 % of the median income. Using a generalized boosted model to estimate the propensity score weights between study groups, this study examined medical utilization, out-of-pocket spending, and the occurrence of catastrophic health expenditure among the study groups.

**Results:**

The findings suggest that the utilization of medical services was not significantly different among the study groups. However, out-of-pocket spending and the occurrence of catastrophic health expenditure were significantly higher in the near-poor group compared to the other two groups.

**Conclusions:**

The study found that the near-poor group was the most vulnerable among the Korean population because of their higher chance of incurring greater out-of-pocket spending and catastrophic health expenditures than is the case among the Medical Aid beneficiary and above-poverty line groups. Health policy needs to take the vulnerability of this near-poor population into account.

## Background

Universal health coverage is intended to allow access to essential health services while providing financial protection from catastrophic health expenditures and subsequent impoverishment due to health care expenses [1]. The public health care system in South Korea is a two-tiered system composed of two programs aimed at providing protection from catastrophic health expenditure and ensuring access to essential health services: National Health Insurance (NHI) and the Medical Aid program. The entire population is covered by one of these two programs. NHI is operated in the form of social insurance with contributions from its members. Medical Aid is a public aid program that guarantees access to needed health services to low-income populations in need of medical assistance. It is comparable to the Medicaid program in the US.

The utilization of medical services is influenced by various factors. Previous studies have indicated that medical service utilization is influenced not only by health status but also by demographic and socioeconomic factors such as age, gender, marital status, education, the particulars of the health care system, and quality of life [2, 3]. Although these factors differ between NHI members and Medical aid beneficiaries [4], Medical Aid beneficiaries suffer greater economic difficulties, higher unemployment, a higher prevalence of chronic disease, and more advanced age compared to NHI members [5]. Due to insufficient ability to care for themselves, Medical Aid beneficiaries face barriers in consuming efficient medical services compared to NHI members [6]. The differences in medical service usage among Medical Aid beneficiaries and NHI members are considered to be closely related to demographic and socioeconomic health-related factors since Medical Aid beneficiaries tend to have higher needs for medical service than health insurance subscribers.

Although, because of strict criteria in the Medical Aid program and the obligatory provider rule, Medical Aid is not covering enough people who are in need of Medical Aid benefits because of their socioeconomic situation. South Korea’s relative poverty rate based on a threshold of below 50 % of national median income was 17.5 % in 2017, but only 3 % of the total population was eligible for Medical Aid in that year [7]. Also, those who meet the income threshold but are excluded from Medical Aid because of the existence of an obligatory provider was estimated at 930,000 in 2015, which accounts for 1.82 % of the total population in that year and equals 56.36 % of the total Medical Aid beneficiaries (1.65 million) in that year [8].

The near-poor are defined under the National Basic Living Security Act as those who are ineligible for public aid programs but who have equivalized disposable household incomes less than 50 % of median household income [9]. The OECD also defines relative poverty as such [10]. The near-poor in South Korea show similar sociodemographic characteristics as Medical Aid beneficiaries, although one study found that the poor not enrolled in Medical Aid included a higher proportion of the elderly and those with less education [11]. Due to these similar characteristics, the near-poor share with Medical Aid beneficiaries, they demonstrate greater medical needs but are experiencing lower medical service utilization and higher levels of unmet needs compared to Medical Aid beneficiaries and non-poor NHI members.

This excluded group is enlisted in NHI. Combined with NHI’s insufficient coverage, the majority of the low-income population remains in a blind spot within the health care system. With the low coverage provided by South Korea’s health insurance, high out-of-pocket spending is blamed for the occurrence of unmet needs among the near-poor caught in this blind spot in the health care system [5]. Excessive out-of-pocket spending caused by the low coverage of NHI makes patients with few financial resources vulnerable to catastrophic health expenditures and subsequent impoverishment. Because of this under-insuring taking place within NHI, it cannot function properly as a primary component of the social safety net protecting citizens from financial crises caused by illness [12, 13].

The characteristics shared by the near-poor with Medical Aid beneficiaries means that the near-poor population tends to experience greater medical needs than the non-poor population. Because they cannot benefit from Medical Aid and are enrolled in NHI, which is criticized for low coverage, the near-poor have a higher chance of facing excessive medical costs and unmet needs. Due to this distinctive characteristic of near-poor NHI members that affect medical utilization, there is a need to distinguish the near-poor population from other NHI members and compare them to each other and to Medical Aid beneficiaries in order to examine the different sociodemographic and policy contexts they inhabit.

Extensive research has been performed on health care utilization and out-of-pocket spending among low-income populations in South Korea. The studies reviewed here indicate that Medical Aid beneficiaries tend to use more medical services but experience less out-of-pocket spending compared to NHI members [14, 15]. However, studies examining health utilization and out-of-pocket spending among the near-poor are limited. Choi (2015) [16] found that poor people not enrolled in Medical Aid had significantly lower medical utilization compared to Medical Aid beneficiaries, and greater healthcare costs as well. However, these studies have only compared the near-poor with Medical Aid beneficiaries and do not include the population above the threshold of 50 % of median income, or else they did not divide the low-income groups according to specific criteria to separate the near-poor out of the low-income population. Studies on the effects of the US Medicaid program among the poor and near-poor showed that the expansion of US Medicaid substantially increased the use of healthcare services, which indicates the possibility of unmet needs among the uninsured near-poor population in the US [17–20].

### Establishment and recent policy changes in the health care system in South Korea

The NHI began in 1977 by covering workers in large corporations. It continuously expanded its coverage to other groups, achieving universal medical coverage after only 12 years [21]. The original form of the Medical Aid program was initiated in 1997 as a part of the South Korean social welfare program and re-envisioned as the Medical Aid program in 2001. The entitlement criterion for Medical Aid is earning less than 40 % of median income. However, if an obligatory provider exists and the obligatory provider’s income exceeds the sum of the median value of the national household income for obligatory providers, and 40 % of the beneficiary’s, the person in question is excluded from Medical Aid enrollment, even if the person’s obligatory provider fails to provide support [7]. Medical Aid beneficiaries are classified as Type I and Type II recipients based on their level of inability to work or incapacitation [22]. Type I beneficiaries are exempted from out-of-pocket payments for any medical utilization covered by the health care system. Type II beneficiaries are required to pay a minimum coinsurance of up to 15 % [1]. Coinsurance for NHI members differs according to the health service and type of hospital involved. For inpatient services, the coinsurance rate is generally up to 20 %. Outpatient services vary with the type of hospital: For clinics, the coinsurance rate is 30 %; hospitals, 40 %; general hospitals, 50 %; and for tertiary hospitals, up to 60 % of the fee [23].

Although South Korea achieved a degree of universal health care with the expansion of NHI in 1989, the regime has been criticized for insufficient benefit coverage. For example, computed tomography was not covered until 1995, magnetic resonance imaging was excluded from the benefits until 2005, and overall coverage reached only 62.7 % in 2017, below the Organization for Economic Cooperation and Development average of 73 % [24].

The South Korean government implemented several policies to increase the coverage of the health insurance system and lessen the burden of health expenditures. A catastrophic health expenditure support program for the population earning below 100 % of the national median income was launched for four major conditions (cancer, cardiovascular and cerebrovascular disease, and rare diseases) in 2013 to control catastrophic medical costs and the occurrence of unmet needs due to high health expenditures in low-income populations [25]. In 2018, this was expanded to cover all inpatient and outpatient services for cancer, cerebrovascular disease, heart disease, rare diseases, severe incurable diseases, and severe burns. Recipients became able to receive up to 20 million won (approximately $18,000) annually for out-of-pocket and uncovered service fees [25]. NHI coverage was extended in 2017 to include coverage for all necessary medical services and control future and existing uncovered services, lower the cost-sharing limit, and prevent catastrophic medical costs [26]. There are several government support programs for the near-poor, but most of these programs are offered in the education and housing sectors. In the health sector, a support program for out-of-pocket spending exists for households below 50 % of the national median income, but the program is limited to chronic diseases only [27].

### Aims

Therefore, this current study aims to examine medical service utilization and out-of-pocket medical spending in near-poor populations by assessing the differences between medical aid beneficiaries and non-poor NHI members. This study analyzes general characteristics by dividing total respondents according to the equivalized disposable household income of 50 % of median income. It then separates the near-poor population from the low-income population according to specific criteria which will be discussed later. In addition, it analyzed medical utilization and out-of-pocket spending and the chance of experiencing catastrophic health expenditure among three separate groups.

## Methods

### Data source

Data were used from the 14th Korea Welfare Panel Study (2019) database, which was conducted by Seoul National University and the Korea Institute for Health and Social Affairs from February 18 through May 21, 2019. The Korea Welfare Panel Study was designed to provide a probability sample of South Korea’s population. The period of the survey was January 1 through December 31, 2018 for flow variables and December 31, 2018 for stock variables.

Among the total of 14,418 individuals from the 14th Korean Welfare Panel Study data, 3,183 individuals were excluded due to being a minor under the age of 18, missing health care program type information, being beneficiaries of free medical treatment for reasons of national merit, and/or as individuals above the poverty line but still beneficiaries of Medical Aid. Finally, 11,235 individuals were selected as subjects of this study.

### Variable definition

#### Defining the Study Group

The study population was categorized into three groups: Medical Aid beneficiaries, the near-poor, and those above the poverty line. The relative poverty line was defined as 50 % of median income by the number of household members in 2018. Near-poor was defined as the population who are below the relative poverty line and enrolled in NHI, those who were subject to National Basic Living Security Aid in 2018 but not enlisted in Medical Aid because an obligatory provider’s income exceeded criteria, or those who were unable to pay the NHI contribution for more than six months and triggered exclusion from NHI benefits. NHI members not classified as near-poor were placed in the above-poverty-line group. The entire population enlisted as Medical Aid beneficiaries were grouped as Medical Aid beneficiaries.

#### Selection and definition of explanatory variables

This study examined two types of medical utilization for 2018: outpatient services and inpatient services. Inpatient services were examined in terms of three variables: hospital visits, hospitalized days, and hospitalized days per visit. For out-of-pocket spending, the Korea Welfare Panel Study includes all out-of-pocket spending, including hospital costs, dental costs, traditional Korean medicine costs, and drug costs. Catastrophic health expenditures are defined as annual out-of-pocket spending exceeding a specified fraction of annual income, which is distinct from high health costs defined simply as those exceeding a predetermined amount [28]. The specified fraction threshold varies between 10 and 40 %: for this study, 10 %, 20 %, 30 %, and 40 % were used as thresholds.

Sociodemographic, health-related, and private insurance-related variables were set as predisposing factors affecting medical utilization and medical spending and included as covariates in regression. For sociodemographic variables, sex, marital status, education status (no completion, below high school diploma, high school diploma, above high school diploma), occupation status (temporary employee, employer, self-employed, unpaid family worker, unemployed or economically inactive, permanent employee), age, and monthly equivalized disposable household income were included. Income was defined as equivalized disposable personal income by dividing household disposable income by the square root of the number of household members to account for differences in household size. For health-related variables, self-perceived health status (healthy, unhealthy), chronic disease states (whether the respondent has at least one chronic disease), depression state, and disorder states were included. Depression state was measured on the CESD-11 scale. Depression status was defined as the sum of CESD-11 questionaries (0–33 points) multiplied by 20/11 being greater than or equal to 16 [29]. Respondents were grouped as having a disorder if any type of mental, kidney, heart, respiratory, liver, physical, speech, facial nerve, brain lesion, visual disturbance, hearing impairment, mental retardation, or intestinal disorder was present. For private insurance-related variables, private insurance subscription status (whether the respondent has at least one type of private insurance) was included.

### Statistical analyses

This study examined the associations of Medical Aid and poverty on health utilization, out-of-pocket spending, and the occurrence of catastrophic health expenditures. Because the decision to use medical services and incur out-of-pocket spending is not random given that an individual’s health status, occupational status, and various other factors influence it, the study applied the model by Rubin (1974) [30]. Most studies use propensity scores to control for two groups, but this study included three groups of interest. To estimate robust propensity scores for three groups with more balance properties, this study applied McCaffrey (2013) ‘s model which uses a machine-learning-driven generalized boosted model (GBM) to estimate propensity score weights among multiple treatment groups [31]. To perform a GBM-estimated propensity score weighting, this study applied Cafalu (2017) ‘s method on the twang package in R run on STATA [32]. Additional information on estimating propensity scores for multiple treatments can be found in McCaffrey (2013) and Cafalu (2017) [31, 32]. Using prior knowledge to include covariates that affect the outcome variable [33], from the predisposing factors mentioned above, this study included sex, age, marital status, education, employment status, income, and private insurance coverage status as covariates to estimate the propensity score. Self-reported health status, chronic disease states, depression states, and disorder states were excluded because there is a chance that the health-related variables might bias the propensity score and lead to bias in the estimated treatment effects.

**Fig. 1:**
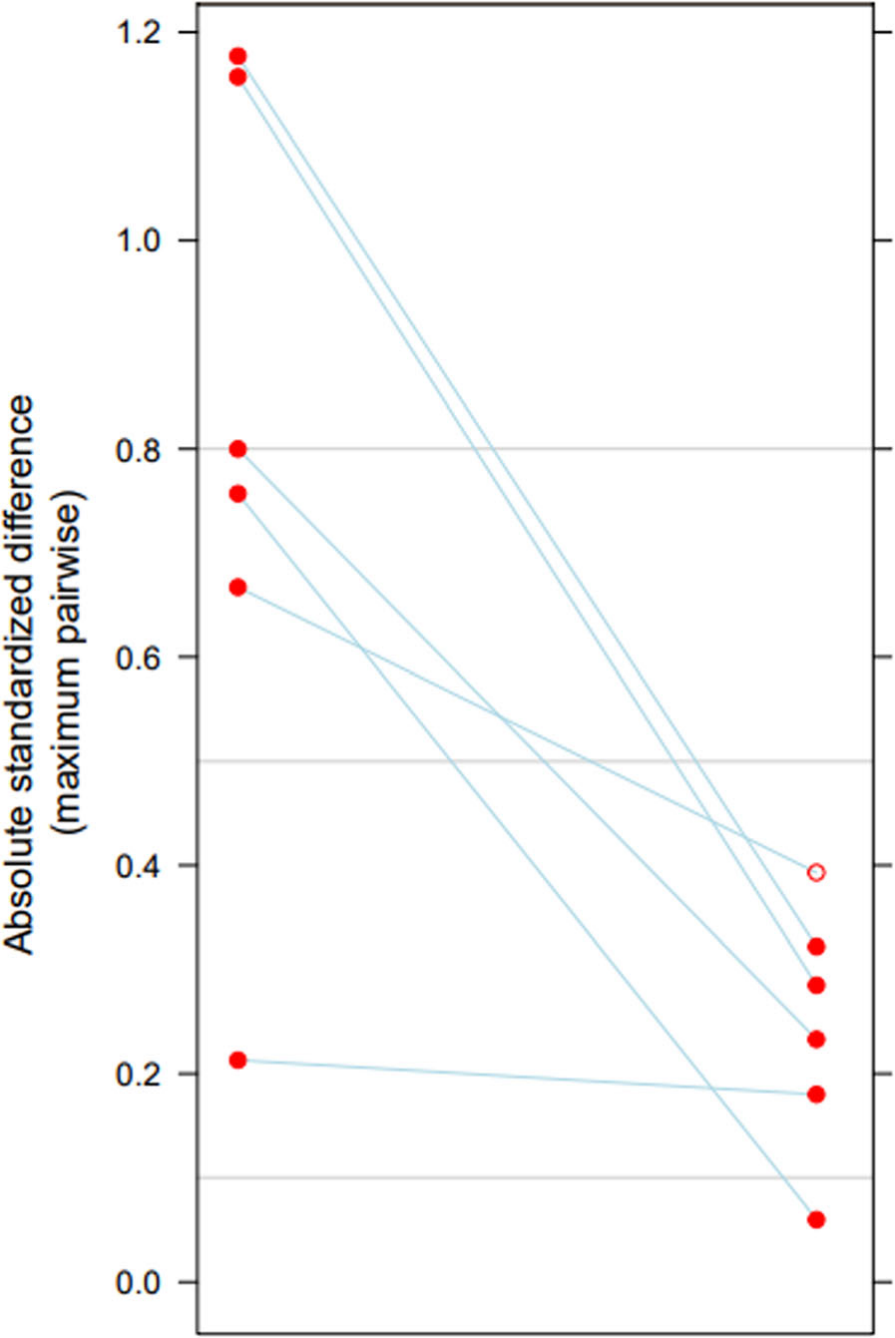
Absolute standardized mean difference for all covariates in before and after propensity score weighting. Right indicates the absolute standardized mean difference before propensity score weighting, and left indicates the absolute standardized mean difference after applying propensity score weighting. Solid circle indicates statistically significant difference; hollow circle indicates statistically insignificant difference

After running the package, the assessment of the weights was checked to make sure that the models were optimized in the balance statistics of interest. It was observed that the balance measures were optimized within 15,000 iterations. The study then compared absolute standardized differences among the included covariates to estimate the propensity score weight (Fig. 1). After propensity score weights were applied, all absolute standardized differences in the covariates included in the estimation had decreased to below 0.4.

This study applied regression with estimated propensity score weights to examine each policy variable’s association with medical utilization and out-of-pocket spending. Poisson and negative binomial models were developed for variables consisting of counts with nonnegative integer values. The negative binomial model is considered a more general model when the assumption of equi-dispersion in the Poisson basic model is not met, by including a random term that reflects the unexplained part in subject differences [34]. To accommodate the overdispersed count nature of outcome variables, this study applied a negative binomial model for outpatient use. The study used a zero-inflated Poisson model (ZIP) for hospital visits and a zero-inflated negative binomial (ZINB) model for hospitalized days and hospitalized days per visit to accommodate unique zero excessive count data structures [35]. Using the ZINB model is more appropriate for zero excessive count data when an assumption of equi-dispersion is not met. However, due to the distribution of hospital visits where zero visits account for 86.15 % (*n* = 9,679) of all respondents and one visit accounts for 77.57 % (*n* = 1207) of the respondents who visited the hospital at least once, the convergence needed to apply the ZINB model could not be met.

Because the out-of-pocket spending data is skewed to the right and was not normally distributed, a generalized linear model (GLM) with a log-link function using a gamma distribution was applied, which is the most suitable option for cost data analysis analyzing both mean and variance functions and adjusting the right-skewed distribution of cost data [36, 37]. The gamma distribution is undefined for values of ‘0’, so an offset of 0.00001 was added to each out-of-pocket spending value in consideration of the users who had no out-of-pocket spending [38].

The occurrence of catastrophic health expenditures was modeled by applying binomial logistic regression to estimate each group’s risk ratio and risk difference. All statistical analyses were performed using Stata ver. 16 (StataCorp, College Station, Texas, USA) and R 4.0.5. This study protocol was approved by the Institutional Review Board of Seoul National University (IRB No. E2010/001–004).

## Results

### Demographic characteristics

Various demographic and socioeconomic characteristics, health status, and occupation status among the three study groups were compared (Table 1). Medical Aid beneficiaries and the near-poor group were found to show similar health and socioeconomic status. However, the near-poor group tends to be more economically active than Medical Aid beneficiaries. The Medical Aid beneficiaries and the near-poor and were substantially older compared to the above-poverty-line group (66.21 versus 72.27 versus 50.27).

**Table 1:** Demographic characteristics of the study population

	Medical Aid beneficiaries		Near-poor group		Above-poverty-line group		Total	
	Frequency	Relative frequency (%)	Frequency	Relative frequency (%)	Frequency	Relative frequency (%)	Frequency	Relative frequency (%)
**N**	616	5.48	2,153	19.16	8,466	75.35	11,235	
**Categorical variables**								
**Female**	384	62.34	1,394	64.75	4,588	54.19	6,366	56.66
**Married**	164	26.62	1,150	53.41	5,554	65.60	6,868	61.13
**Education**								
∈no diploma	110	17.86	493	22.90	245	2.89	848	7.55
∈below high school diploma	328	53.25	1,221	56.71	2,021	23.87	3,570	31.78
∈high school diploma	129	20.94	303	14.07	2,665	31.48	3,097	27.57
∈above high school diploma	49	7.95	136	6.32	3,535	41.76	3,720	33.11
**Occupation**								
∈ permanent employee	2	0.32	15	0.70	2,319	27.39	2,336	20.79
∈temporary employee	52	8.44	309	14.35	1,891	22.34	2,252	20.04
∈ employer, self-employed, unpaid family worker	11	1.79	302	14.03	1,161	13.71	1,474	13.12
∈ unemployed, economically inactive	551	89.54	1,527	70.92	3,095	36.56	5,173	46.04
**Reporting bad health**	358	58.12	1,025	47.61	1,227	14.49	2,610	23.23
**Chronic disease**	527	85.55	1,820	84.53	3,971	46.91	6,318	56.23
**Disabled**	115	18.67	170	7.90	265	3.13	550	4.90
**Private insurance subscription**	123	19.97	468	21.74	6,499	76.77	7,090	63.11
**Depression**	276	44.81	649	30.14	782	9.24	1,707	15.19
**Continuous variables**								
**Age**	66.21 (mean)	16.60 (SD)	72.27 (mean)	13.02 (SD)	50.27 (mean)	17.65 (SD)	55.30 (mean)	19.02 (SD)
**Monthly income (one million won)**	0.92 (mean)	0.31 (SD)	0.81 (mean)	0.53 (SD)	3.13 (mean)	2.11 (SD)	2.56 (mean)	2.09 (SD)

### Differences in health utilization among study groups

Both Medical Aid beneficiaries and the above-poverty-line group had no significant difference in outpatient and inpatient utilization compared to the near-poor group (Table 2). Respondents who had the chronic disease were estimated to have 238 % more outpatient visits, 36 % less chance of having no hospitalized days, and 50 % more hospitalized days in all three groups. Respondents who reported having poor health were estimated to have 53 % more outpatient visits and 36 % less chance of zero hospitalized days, 50 % more hospitalized days, and 78 % more hospitalized days per visit compared to those who did not report poor health.

**Table 2:** Medical utilization among study groups

	Outpatient visits		Hospital visits				Hospitalized days				Hospitalized days per visit			
	Negative binominal		Poisson		Logit		Negative binominal		Logit		Negative binominal		Logit	
	*β*	SE	*β*	SE	*β*	SE	*β*	SE	*β*	SE	*β*	SE	*β*	SE
**Study group**														
∈Near-poor	-	-	-	-	-	-	-	-	-	-	-	-	-	-
∈ Medical Aid	0.20*	0.11	0.15	0.73	0.95	2.23	-0.26	0.16	0.42*	0.23	-0.17	0.16	0.43*	0.23
∈Above-poverty	-0.03	0.08	0.18	1.02	0.41	2.99	-0.15	0.12	-0.01	0.18	-0.15	0.12	-0.02	0.18
**Female**	0.30***	0.08	-0.34	0.48	-0.60	1.26	-0.19*	0.12	-0.02	0.20	-0.22*	0.12	-0.02	0.20
**Age**	0.02***	0.00	-0.02**	0.01	-0.04**	0.02	0.01	0.01	0.00	0.01	0.02***	0.01	0.00	0.01
**Married**	0.03	0.07	-0.36	0.60	-0.07	1.84	-0.21*	0.13	0.32*	0.19	-0.18	0.12	0.32*	0.19
**Education**														
∈no diploma	-	-	-	-	-	-	-	-	-	-	-	-	-	-
∈below high school diploma	0.03	0.07	0.29	0.31	0.06	0.63	-0.06	0.15	-0.17	0.16	-0.16	0.16	-0.18	0.16
∈high school diploma	0.00	0.12	-0.06	0.33	-0.78	0.68	-0.07	0.23	-0.23	0.26	-0.19	0.22	-0.24	0.26
∈above high school diploma	-0.04	0.14	-0.40	0.42	-1.82*	1.09	-0.22	0.29	-0.33	0.25	-0.22	0.27	-0.32	0.25
**Occupation**														
∈ permanent employee	-	-	-	-	-	-	-	-	-	-	-	-	-	-
∈temporary employee	-0.36*	0.19	-1.06	1.31	-0.64	2.52	-0.68**	0.32	0.77*	0.47	-0.75**	0.34	0.75	0.47
∈ employer, self-employed, unpaid family worker	-0.30	0.19	-0.71	3.56	-1.37	10.44	-0.51*	0.31	0.27	0.48	-0.65**	0.34	0.25	0.48
∈ unemployed, economically inactive	-0.26	0.18	-0.07	1.06	-0.54	1.92	-0.27	0.30	-0.01	0.44	-0.45	0.33	-0.04	0.44
**Monthly income (increase of one million won)**	-0.01	0.02	-0.07**	-0.03	0.03	0.06	-0.02	0.03	0.09	0.06	-0.02	0.02	0.09	0.06
**Self-reporting poor health**	0.43***	0.06	1.09	1.47	-0.32	3.28	0.69***	0.13	-1.21***	0.19	0.58***	0.13	-1.22***	0.19
**Chronic disease**	1.22***	0.08	0.90	1.12	0.80	3.48	0.41**	0.18	-0.46**	0.21	0.30	0.19	-0.47**	0.21
**Disabled**	0.00	0.07	-0.10	0.33	-0.10	0.50	0.27	0.21	0.18	0.18	0.08	0.18	0.17	0.18
**Private insurance subscription**	0.12	0.07	-0.22	1.01	-1.05	1.98	0.02	0.16	-0.31**	0.14	0.10	0.14	-0.30**	0.14
**Depression**	0.21***	0.08	0.59	1.58	0.54	3.64	0.33**	0.14	-0.17	0.18	0.16	0.13	-0.18	0.18

*** *p* < 0.001 ** *p* < 0.05 * *p* <0.1

### Differences in out-of-pocket spending and chance of experiencing catastrophic health expenditure among the study groups

For out-of-pocket spending, the Medical Aid beneficiaries showed 66 % less out-of-pocket spending, but the above-poverty group incurred 30 % more out-of-pocket spending compared to the near-poor group (Table 3). In terms of the chance of experiencing catastrophic health expenditures, both Medical Aid beneficiaries and the above-poverty group were estimated to have a significantly less chance of experiencing catastrophic health expenditures at all thresholds compared to the near-poor group.

**Table 3:** Out-of-pocket (OOP) spending and occurrence of catastrophic health expenditures (CHE) among study groups

	OOP spending		CHE (10%)		CHE (20%)		CHE (30%)		CHE (40%)	
	Log-link GLM		Logit		Logit		Logit		Logit	
	*β*	SE	*OR*	SE	*OR*	SE	*OR*	SE	*OR*	SE
**Study group**										
∈Near-poor	-	-	-	-	-	-	-	-	-	-
∈Medical Aid	-1.09***	0.13	0.21***	0.06	0.20***	0.06	0.10***	0.02	0.07***	0.02
∈Above-poverty	0.27***	0.07	0.52**	0.15	0.39**	0.19	0.36**	0.16	0.31***	0.10
**Female**	-0.06	0.07	0.89	0.14	1.01	0.20	0.87	0.20	0.87	0.27
**Age**	-0.01***	0.00	1.00	0.00	1.01	0.01	1.00	0.01	1.00	0.01
**Married**	0.36***	0.08	1.16	0.18	1.61**	0.34	1.00	0.23	0.85	0.26
**Education**										
∈no diploma	-	-	-	-	-	-	-	-	-	-
∈below high school diploma	0.02	0.08	1.10	0.15	1.20	0.17	1.26	0.18	1.10	0.18
∈high school diploma	-0.09	0.11	0.66**	0.14	0.85	0.21	1.07	0.29	0.90	0.31
∈above high school diploma	-0.29**	0.12	0.52***	0.14	0.51**	0.18	0.87	0.27	0.71	0.25
**Occupation**										
∈ permanent employee	-	-	-	-	-	-	-	-	-	-
∈temporary employee	-0.26	0.18	0.54	0.24	0.27**	0.15	0.27**	0.17	0.17***	0.11
∈ employer, self-employed, unpaid family worker	-0.18	0.19	0.48*	0.22	0.33*	0.20	0.66	0.43	0.38	0.26
∈ unemployed, economically inactive	-0.04	0.17	0.71	0.31	0.50	0.29	0.88	0.53	0.48	0.29
**Monthly income (increase of one million won)**	0.03*	0.02	0.74*	0.14	0.71	0.25	0.79	0.26	0.81	0.17
**Self-reporting poor health**	0.43***	0.08	2.17***	0.33	1.88***	0.28	2.39***	0.32	2.56***	0.45
**Chronic disease**	0.21**	0.09	1.46**	0.28	1.16	0.27	1.82***	0.41	1.75**	0.51
**Disabled**	-0.06	0.10	0.88	0.16	0.94	0.18	0.84	0.18	0.94	0.19
**Private insurance subscription**	0.18***	0.07	1.04	0.14	1.17	0.18	1.11	0.14	1.05	0.16
**Depression**	0.25**	0.12	1.46	0.35	1.72**	0.45	1.48***	0.22	1.65***	0.29

*** *p* < 0.001 ** *p* < 0.05 * *p* <0.1

### Predicted value of health service utilization and out-of-pocket spending and likelihood of experiencing catastrophic health expenditure among study groups

The predicted values of health service utilization were in ascending order of Medical Aid beneficiaries, the near-poor, and the above-poverty line group (Table 4). For inpatient services, all three variables were also in ascending order of the near-poor, Medical Aid beneficiaries, and the above-poverty line group. The predicted value of out-of-pocket spending rose from the above-poverty line group to the near-poor group to the Medical Aid beneficiaries group. The likelihood of incurring catastrophic health expenditures was in the ascending order of the near-poor, Medical Aid recipients, and the above-poverty line group at all four thresholds.

**Table 4:** Predicted value of health service utilization and out-of-pocket spending, chance of occurrence of catastrophic health expenditure

	Outpatient visits		Hospital visits		Hospitalized days		Hospitalized days per visit			
	Mean	SD	Mean	SD	Mean	SD	Mean	SD		
**Medical Aid**	36.16	18.38	0.33	0.24	5.63	4.67	3.99	3.12		
**Near-poor**	29.82	14.66	0.34	0.27	6.97	6.74	4.98	4.39		
**Above-poverty**	13.71	11.81	0.15	0.16	1.92	3.16	1.52	2.16		
	OOP spending		CHE (10%)		CHE (20%)		CHE (30%)		CHE (40%)	
	Mean	SD	Mean	SD	Mean	SD	Mean	SD	Mean	SD
**Medical Aid**	80.04	28.91	0.32	0.13	0.16	0.08	0.06	0.03	0.02	0.01
**Near-poor**	226.16	84.77	0.65	0.14	0.46	0.16	0.33	0.15	0.22	0.12
**Above-poverty**	299.01	96.43	0.23	0.14	0.10	0.08	0.05	0.05	0.02	0.03

## Discussion

This study found that the near-poor group was the most vulnerable group among the South Korean population in terms of their greater out-of-pocket spending and higher chance of incurring catastrophic health expenditures. There were no significant differences in medical utilization between the near-poor and either Medical Aid beneficiaries or the above-poverty group after controlling for potential bias. In addition, the near-poor were found to incur more out-of-pocket spending than Medical Aid beneficiaries and to have a significantly higher chance of experiencing catastrophic health expenditures at all thresholds compared to the two other groups. These results demonstrate that the near-poor group could be considered the most vulnerable population based on medical service utilization and out-of-pocket spending. This assumption is in agreement with the results reported by several other studies. Medical Aid beneficiaries were found to use more inpatient and outpatient services, incurred less out-of-pocket spending, and had a lower chance of experiencing catastrophic health expenditures compared to NHI members [39]. A study that defined the near poor as people not enrolled in Medical Aid but with an income less than 120 % of the minimum cost of living found that Medical Aid beneficiaries experienced significantly lower health care costs and proportion of out-of-pocket spending to income compared to the poor not enrolled in Medical Aid [11]. Studies conducted in the US showed that the most important factor in determining whether people are obtaining sufficient medical service is being uninsured with low income [40, 41]. Studies on the effect of the US Medicaid program among the poor and near-poor have shown that the uninsured experienced difficulties obtaining medical care and that the expansion of US Medicaid lowered out-of-pocket costs [42–44]. However, contrary to previous findings, this study indicates that medical utilization did not differ among the three groups. This might demonstrate the significant association of the expansion of the catastrophic health expenditure support program in 2018 and the overall NHI coverage expansion that has been taking place since 2017 on medical utilization. Furthermore, the current administration implemented an NHI coverage expansion in 2017 by alleviating the uncovered services cost burden [45]. Because previous research was based on data from before 2016, the findings do not reflect these recently implemented policies affecting the medical utilization of the near-poor. Further study is needed to examine the outcome of these latest policy changes on medical utilization among the near-poor in South Korea.

Although medical utilization showed no significant difference for the above-poverty and the near-poor groups, out-of-pocket spending in the above-poverty group was greater compared to among the near-poor. There is a chance that the recently implemented out-of-pocket spending threshold restricting out-of-pocket spending to 10 % of annual income for the bottom 50 % income group has influenced the out-of-pocket spending of the near-poor in terms of association [45]. Also, South Korea’s NHI has been criticized for insufficient coverage, meaning many medical services are perceived as going uncovered. Due to economic difficulties, the near-poor have a limited capacity to use uncovered services compared to the non-poor, resulting in uncovered health care utilization and out-of-pocket spending being skewed toward the better-off [46–48]. At the same time, the better-off are more likely to be subscribers to private health insurance, impacting their out-of-pocket costs [49].

The presence of chronic disease was significantly associated with greater outpatient and inpatient use among the three study groups. Chronic disease has been confirmed in several studies as a significant factor in determining outpatient service use, such as in terms of the number of outpatient visits [50]. Because the average age of this study population is high, the presence of chronic disease is likely associated with higher inpatient use due to a lack of proper self-management [51, 52]. Additional focus is required on chronic disease prevention by empowering the population through strengthened education. Self-management programs must be supported in order to mitigate hospitalization due to chronic disease. In addition, perceived negative health status was associated with greater outpatient and inpatient use. Perceived negative health status can lead to poor physical health and greater social isolation [53]. Thus, self-evaluated health status should be considered in the development of health promotion programs for both Medical Aid beneficiaries NHI members.

South Korea has regularly implemented expansions of NHI coverage and pursued the reduction of copayments and the implementation of support programs for catastrophic health expenditures among the poor population to ensure proper health care use and prevent impoverishment due to health care costs. Despite these efforts, several studies, including this study, have demonstrated that the near-poor population remains unprotected from the occurrence of catastrophic health expenditures. Moreover, previous studies have found that the near-poor population experiences higher unmet needs compared to Medical Aid beneficiaries [54]. An expansion of Medical Aid could be considered an alternative for alleviating this burden and ensuring the provision of essential health services among the near-poor. Lee (2020) [15] found that people who shifted from NHI to Medical Aid raised their number of outpatient visits without increasing out-of-pocket spending. A more focused policy for populations in this blind spot within the health care system, including their perceived health status and chronic disease, is required to ensure the provision of essential health services to the near-poor group.

This study has certain limitations and strengths. The findings may not be generalizable to other countries with divergent medical utilization and out-of-pocket spending programs. Second, the issue of supply-induced demand among Medical Aid beneficiaries could not be resolved. Third, although propensity score weighting was used to adjust for potential bias, several factors that could influence medical utilization and out-of-pocket spending could not be examined due to a lack of data. Finally, given the limits of this data, the use of uncovered medical services could not be identified because medical services were not categorized as covered or uncovered. Because several high-quality medical services offered in South Korea are not covered by either Medical Aid or NHI, the quality of medical service that respondents experienced could not be verified. Future research should examine the various factors that may influence medical utilization and out-of-pocket spending, including variables such as unmet needs, health service quality, and service accessibility.

The strengths of this study include its analysis of socioeconomic and health-related factors and the use of different statistical methods to accommodate the unique characteristics of outcome variables and minimize potential bias. In addition the medical utilization and out-of-pocket spending of an above-poverty-line group not included in previous studies were examined.

## Conclusions

This study found that the near-poor population showed no significant difference in medical utilization compared to the Medical Aid and above-poverty-line groups, but they incurred greater out-of-pocket spending and were exposed to a higher likelihood of incurring catastrophic health expenditures. This result indicates that the near-poor group is the most vulnerable within South Korea’s population. Health policy needs to take this vulnerability of the near-poor population into account along with several factors, such as chronic disease and perceived health status, that is associated with medical use and cost in order to ensure essential services and provide protection from impoverishment by health care costs.

## Data Availability

The data set analyzed for the current study is available as part of the Korea Welfare Panel Study, [https://www.koweps.re.kr/].

## Abbreviations

NHI: National Health Insurance
CESD: Center for Epidemiological Studies-Depression
GBM: Generalized boosted model
ZIP: Zero-inflated Poisson
ZINB: Zero-inflated negative binomial
GLM: Generalized linear model
